# Response to winter pressures in acute services: analysis from the Winter Society for Acute Medicine Benchmarking Audit

**DOI:** 10.1186/s12913-021-07355-7

**Published:** 2022-01-02

**Authors:** Catherine Atkin, Thomas Knight, Chris Subbe, Mark Holland, Tim Cooksley, Daniel Lasserson

**Affiliations:** 1 Birmingham Acute Care Research Group, Institute of Inflammation and Ageing, University of Birmingham, Edgbaston, Birmingham, B15 2GW UK; 2 Department of Acute Medicine, Sandwell and West Birmingham NHS Trust, City Hospital, Birmingham, B18 7QH UK; 3 School of Medical Sciences, Bangor University & Consultant Acute, Respiratory & Critical Care Medicine, Ysbyty Gwynedd, Bangor, LL57 2PW UK; 4 Clinical and Biomedical Sciences, Faculty of Health and Wellbeing, University of Bolton, Bolton, BL3 5AB UK; 5 Department of Acute Medicine, Manchester University NHS Foundation Trust, Manchester, M23 9LT UK; 6 Department of Acute Medicine, The Christie, Manchester, M20 4BX, UK; 7 Division of Health Sciences, Warwick Medical School, University of Warwick, Coventry, CV4 7AL UK; 8 Division of Acute General Medicine, Oxford University Hospitals NHS Foundation Trust, Headington, Oxford, OX3 9DU UK

**Keywords:** Acute medicine, Winter pressure, Service planning, Same day emergency care

## Abstract

**Background:**

There is increased demand for urgent and acute services during the winter months, placing pressure on acute medicine services caring for emergency medical admissions. Hospital services adopt measures aiming to compensate for the effects of this increased pressure. This study aimed to describe the measures adopted by acute medicine services to address service pressures during winter.

**Methods:**

A survey of acute hospitals was conducted during the Society for Acute Medicine Benchmarking Audit, a national day-of-care audit, on 30th January 2020. Survey questions were derived from national guidance.

Acute medicine services at 93 hospitals in the United Kingdom completed the survey, evaluating service measures implemented to mitigate increased demand, as well as markers of increased pressure on services.

**Results:**

All acute internal medicine services had undertaken measures to prepare for increased demand, however there was marked variation in the combination of measures adopted. 81.7% of hospitals had expanded the number of medical inpatient beds available. 80.4% had added extra clinical staff. The specialty of the physicians assigned to provide care for extra inpatient beds varied. A quarter of units had reduced beds available for providing Same Day Emergency Care on the day of the survey. Patients had been waiting in corridors within the emergency medicine department in 56.3% of units.

**Conclusion:**

Winter pressure places considerable demand on acute services, and impacts the delivery of care. Although increased pressure on acute hospital services during winter is widely recognised, there is considerable variation in the approach to planning for these periods of increased demand.

## Introduction

Urgent and acute hospital services within the United Kingdom are placed under growing strain year on year, with ongoing increases in the number of patients attending and admitted to acute hospitals [1]. The demand on services rises during winter months. This ‘winter pressure’ is well recognised within the National Health Service (NHS) [2], and has multiple underlying causes, with seasonal variations in disease and infection, including respiratory illnesses [3, 4], and increased length of stay for patients admitted to hospital during winter [2]. Seasonal variations in disease incidence and severity are seen across multiple conditions [5–7], and all-cause mortality has been shown to increase in winter [8].

Medical emergencies are the most frequent cause of unplanned admission to acute hospitals; patients presenting with medical problems are referred from the emergency medicine department or from the community through primary care services for assessment and treatment by the acute internal medicine team [9]. These patients may be acutely unwell, needing rapid diagnosis and initiation of treatment [10]. It is therefore vital that patients admitted with medical emergencies are identified, assessed and reviewed by appropriate clinical decision makers as soon as possible [11], and it is equally important that these processes are maintained through times of increased pressure.

Although there are some national recommendations on how to prepare for winter pressures, including the development of local escalation plans and increased staffing in times of pressure [2, 12], there is little evidence evaluating the measures that are currently used within acute medical services.

The Society for Acute Medicine Benchmarking Audit (SAMBA) aims to provide a national comparison of performance against key clinical quality indicators in acute medicine services [13], and evaluate processes of acute care on a national level, assessing variations in service design and delivery between centres. Through SAMBA, acute medicine services nationally were surveyed regarding their winter pressure preparations and markers of pressure within their departments.

## Methods

The winter round of SAMBA 2020 took place on 30th January 2020. Participation in SAMBA is voluntary, with individual units registering locally at each site, and nationally through an online portal via the Society for Acute Medicine (SAM). Registration is available to all hospitals in the United Kingdom who accept unplanned medical admissions; non-acute and community hospitals are excluded. Multiple units can register per hospital and per NHS Trust. This includes acute medical units (AMUs), dedicated units that provide the rapid assessment, close monitoring and access to specialist services necessary for the management of acutely unwell medical patients, and ambulatory emergency care (AEC) units, where same day emergency care (SDEC) is provided by the medical team for more stable patients, aiming to facilitate diagnosis and management without admission to an inpatient bed.

SAMBA collects data on unit structure and service delivery, including the number of inpatient beds available on the acute medical unit and in total within the hospital. Questions are developed through a national multi-professional forum, including physicians, nurses and pharmacists, and are informed by national guidance, health care policy and standards set by professional bodies. During Winter SAMBA [14], additional questions were asked regarding changes made to service delivery during the winter period, based on reports and guidance from the Care Quality Commission [12], NHS Improvement [2], NHS England [15] and Health Education England [16]. SAMBA is registered with the Healthcare Quality Improvement Partnership (HQIP https://www.hqip.org.uk). The North-West Wales Ethics Committee confirmed that the process for SAMBA described here did not require formal ethical review.

The included questions covered two areas: planned changes implemented prior to the day of data collection, and response to pressure on the day of the survey (Table 1). Units were asked to report their Operational Pressure Escalation Level (OPEL) on the day of the audit; these levels are part of a national framework intended to guide responses to fluctuates in service pressure and demand.

**Table 1:** Questions asked during survey regarding acute services

**Questions regarding planned changes (before day of survey):**	
• Does your Trust have an escalation plan?	
• Have extra medical beds been created for winter pressures? *e.g. an escalation ward*	
• If you have an escalation ward, which team provides daytime medical cover for these patients? ○ Acute medicine, dedicated medical team, general medicine team with responsibilities for other wards, medical specialties, other (tick all that apply)	
• Which team provides daytime medical cover for patients who are medical outliers on non-medical wards? ○ Acute medicine, dedicated medical team, general medicine team with responsibilities for other wards, medical specialties, other (tick all that apply)	
• Have doctors in training been moved to help with increased pressures?	
• Have extra clinical staff been added to help with winter pressures?	
• Have extra non-clinical staff been added to help with winter pressures?	
• In times of pressure, is your AEC ever bedded?	
• Have teaching sessions been cancelled due to winter pressures? ○ Some, all or none	
**Questions regarding pressure on the day of the survey:**	
• What OPEL level was your Trust on?	
• Had your Trust started using their escalation plan?	
• Were elective surgeries cancelled in the week up to and including the day of the survey? ○ Some, all or none	
• Was your AEC area bedded?	
• Did your hospital have any bed closures affecting the number of beds? e.g. *due to norovirus or flu*	
• Were ambulances diverted away from your hospital’s emergency department at any time?	
• Did patients have to wait in emergency department corridors?	
• At 16:00, were there patients in AMU who did not have an AMU bed?	
• If yes, how many?	

*AEC* Ambulatory Emergency Care, *OPEL* Operational Pressures Escalation Levels, *AMU* Acute Medical Unit

Descriptive statistics were calculated using Microsoft Excel and IBM SPSS statistics version 25.

## Results

105 hospitals participated in the audit, with 93 hospitals completing the questions relating to winter pressures (88.6%). The median number of inpatient beds for participating hospitals was 505 (IQR 397–763, range 76–1700); median number of AMU beds was 39 (IQR 29–52, range 13–93). The median number of patients seen by each unit on the day of the survey was 51 (IQR 36–73, range 15–129).

### Planned changes

**Fig. 1:**
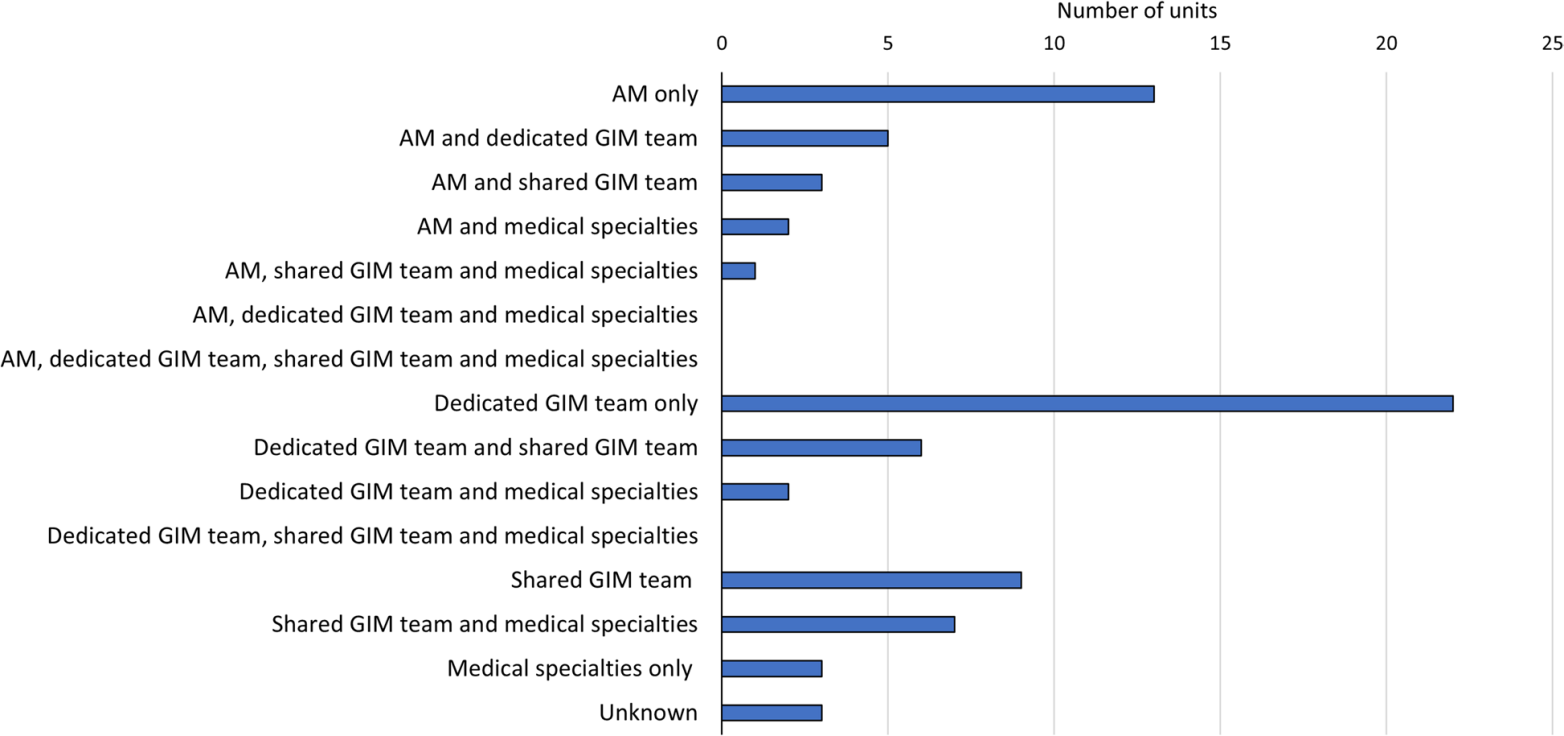
Teams staffing extra beds for winter pressures. AM: Acute Medicine; GIM: General Internal Medicine. Shared GIM teams have responsibility for wards other than the extra beds described here

Only one unit recorded that they did not have an escalation plan. 81.7% of hospitals (76/93) had created extra medical beds for winter pressures. The clinical teams responsible for providing medical care for these beds are shown in Fig. 1.

**Fig. 2:**
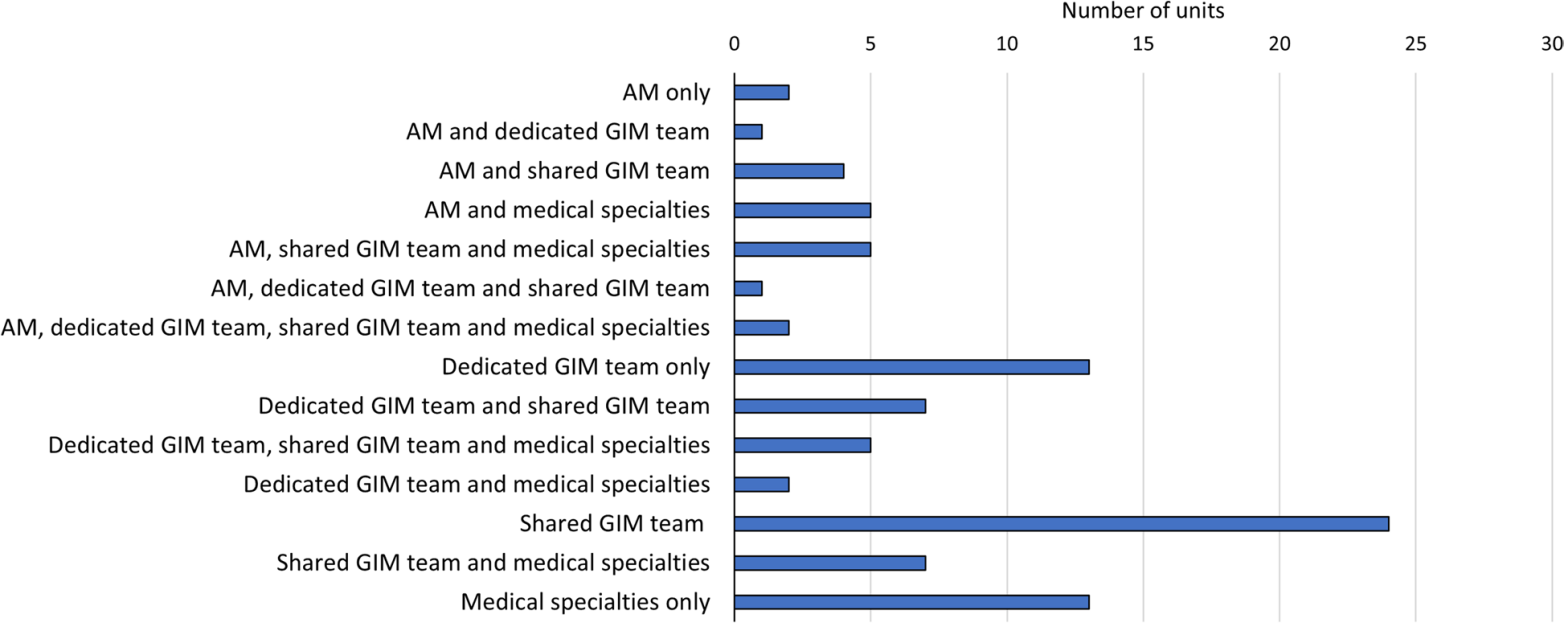
Teams providing cover for medical patients on non-medical wards. AM: Acute Medicine; GIM: General Internal Medicine. Shared GIM teams have responsibility for wards other than the medical patients on non-medical wards described here

The team providing care for medical outliers (medical patients on non-medical wards) during routine daytime hours varied (Fig. 2); most commonly this was provided by a general internal medicine team who also had responsibility for other wards (26.4%, 24/91 units).

53.3% of units (49/92) reported that the area where they usually provide SDEC would never be converted to inpatient beds in times of increased pressure.

**Fig. 3:**
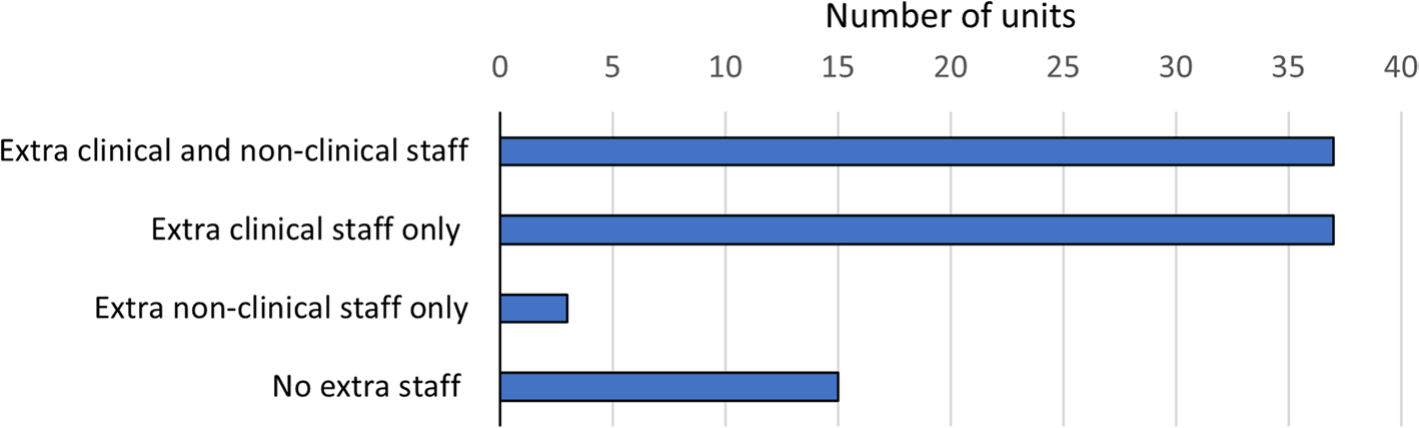
Additional staffing for winter pressures

Extra staff had been added to help with winter pressures in 83.7% of units (77/92), with extra clinical staff added in 80.4% (74/92) and extra non-clinical staff in 43.5% (40/92, Fig. 3). Doctors in training grades had been moved from the area where they were originally allocated to help with pressures in 43.0% of hospitals (40/93).

Teaching sessions had been cancelled due to winter pressures in 53.8% of units (50/93), although no units reported cancelling all teaching sessions.

There was no statistically significant difference between the size of AMU who undertook any planned change compared to those who did not (Table 2). Hospitals that had moved junior doctors in training due to winter pressures had a higher median number of total hospital inpatient beds (596 vs 453, Mann Whitney U test *p* = 0.002). Those who had moved junior doctors in training due to winter pressures saw a higher median number of patients on the day of the survey (median 68 vs 46, *p* = 0.004), as did those who had added extra clinical staff (median 54 vs 39, *p* = 0.008).

**Table 2:** Comparison of planned services changes and measures of pressure to unit size

		Measures of hospital size					
		AMU beds		Inpatient beds		Medical patients admitted on day of survey	
		Median (IQR)	*p* value	Median (IQR)	*p* value	Median (IQR)	*p* value
**Planned changes**							
Extra medical beds created for winter pressures	Y	40 (30–52)	0.22	529 (407–763)	0.20	51 (36–75)	0.34
	N	33 (24–51)		435 (325–648)		39 (33–70)	
Doctors in training moved to help pressure	Y	42 (28–56)	0.18	596 (456–846)	**0.002**	68 (45–79)	**0.004**
	N	37 (29–50)		453 (331–677)		46 (33–64)	
Extra clinical staff	Y	40 (31–52)	0.092	516 (415–756)	0.12	54 (36–76)	**0.008**
	N	31 (24–49)		430 (275–789)		39 (27–50)	
Extra non-clinical staff	Y	38 (28–52)	0.91	544 (418–794)	0.19	60 (36–76)	0.17
	N	39 (30–51)		470 (395–669)		48 (33–69)	
SDEC area ever converted to inpatient beds	Y	39 (29–52)	0.69	500 (359–669)	0.34	51 (33–72)	0.35
	N	40 (30–52)		525 (430–789)		49 (37–76)	
Any teaching sessions cancelled	Y	36 (28–50)	0.22	542 (431–792)	0.24	52 (37–74)	0.39
	N	40 (33–53)		480 (362–680)		46 (34–72)	
**Measures of pressure**							
Utilising escalation plan	Y	38 (29–52)	0.23	525 (430–792)	0.40	51 (36–75)	0.81
	N	47 (40–50)		460 (358–647)		53 (34–73)	
Any elective surgery cancelled	Y	36 (26–51)	0.30	525 (415–767)	0.68	49 (36–69)	0.36
	N	40 (31–53)		470 (405–767)		53 (34–77)	
SDEC area converted to inpatient beds on day of survey	Y	38 (28–52)	0.72	505 (384–732)	0.77	52 (33–72)	0.44
	N	39 (30–51)		505 (413–786)		50 (37–76)	
Bed closures	Y	46 (33–51)	0.63	564 (450–756)	0.72	54 (49–80)	0.20
	N	38 (29–52)		503 (384–792)		49 (34–73)	
Patients waiting in Emergency Department corridor	Y	36 (4–53)	0.46	532 (382–792)	0.54	62 (37–80)	0.05
	N	41 (33–50)		485 (415–698)		47 (34–63)	
Patients without allocated inpatient bed in AMU at 16:00	Y	34 (27–52)	0.49	503 (451–779)	0.30	63 (42–79)	0.09
	N	40 (30–51)		503 (368–741)		49 (33–72)	
OPEL level	1	32 (27–50)	0.78*	403 (318–480)	0.24*	33 (26–66)	0.18*
	2	36 (30–45)		539 (429–808)		46 (33–67)	
	3	42 (29–52)		525 (400–782)		52 (38–73)	
	4	43 (31–53)		597 (458–805)		71 (47–82)	

Unit size assessed by number of acute medical inpatient beds, total inpatient beds and number of acute medical inpatient admissions on the day of the survey. *AMU* Acute Medical Unit, *SDEC* Same Day Emergency Care, *OPEL* Operational Pressures Escalation Levels. Comparisons performed using Mann-Whitney U test; *performed using Kruskal-Wallis test

Amongst the 93 participating units, there were 91 individual combinations of responses to the questions regarding planned changes for winter pressures.

### Measures of pressure on the day of survey

84.9% of units (73/86) had started using their escalation plan prior to the day of the survey. Comparing OPEL level on the day of the survey, 8 units were level 1 (9.4%), 22 were level 2 (25.9%), 43 units were level 3 (50.6%), and 12 units were at the highest level (level 4, 14.1%)

15.9% of hospitals (14/88) had bed closures, reducing the number of beds available. Two hospitals had emergency ambulances diverted away from the emergency medicine department at any point of the day of the survey.

45.3% of units had cancelled some elective surgical procedures in the week leading up to the survey (39/86); all elective surgery had been cancelled in one unit.

An area designated for providing AEC was used for inpatient beds on the day of the survey in 22 units, equating to 51.2% of the units where this AEC area was ever converted to inpatient beds, and 25.0% of units overall.

**Fig. 4:**
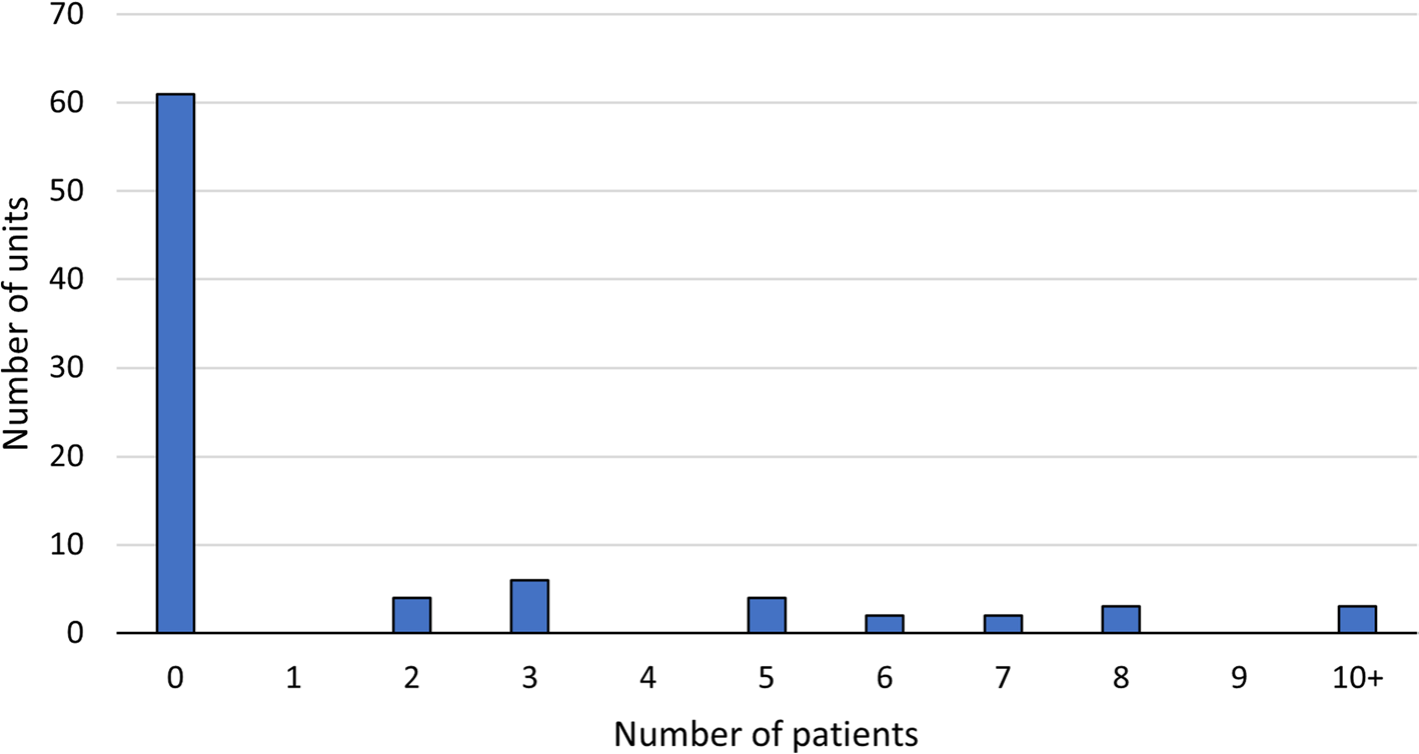
Number of patients without an allocated bed. Number of patients present in the Acute Medical Unit at 16:00 on the day of the survey who did not have an allocated bed available

In 56.3% of units (49/87) patients had needed to wait in corridors within the emergency medicine department on the day of the survey, and 27.9% of units had one or more patients in AMU at 16:00 who did not have an allocated AMU inpatient bed available (Fig. 4).

The number of AMU beds, total number of inpatient beds and number of patients seen on the day of the survey were not significantly different comparing those who reported each measure of pressure on the day of the survey and those who did not.

## Discussion

All acute internal medicine services surveyed had undertaken measures to plan for increased demand during the winter. Some measures were adopted widely – more than 80% of hospitals had expanded the number of medical inpatient beds available, and 80.4% had added extra clinical staff. There was considerable variation in the physician teams used to provide senior physician input for medical patients, for additional inpatient beds that had been added due to winter pressures and for medical inpatients on non-medical inpatient wards. Approaches varied from dedicated teams specialising in general internal medicine, to shared responsibility across physicians specialising in acute medicine, other medical specialties and consultants with responsibilities for other general internal medical wards.

There is a striking variability in the range of approaches to winter pressure planning. From the 93 centres included, there were 91 different responses to the nine questions describing preparations for winter pressure.

The adoption of most planned measures did not seem to vary based on hospital size, although larger hospitals more commonly moved doctors in training. Although hospital size may affect the ability to introduce some planning measures, as there may be greater resource available to allocate to extra beds or to areas of pressure, there was no clear relation to hospital size for most approaches described here. The variability seen suggests decisions made in planning may be more complex, involving multiple factors, and cannot be easily categorised or evaluated without more detailed information.

Many of the units who took part in this survey were under pressure. A quarter of units reported their ambulatory emergency care facilities being used for inpatient beds. This may reduce the ability of acute medical units to deliver SDEC, which in turn may worsen pressure on services and demand for inpatient bed availability. These areas providing SDEC should therefore be protected, including during periods of winter pressure [17].

On the day of the survey, more than half of units reported that patients had to wait in emergency medicine department corridors, and more than a quarter of units had patients in the acute medical unit without an allocated inpatient bed. Providing care in inappropriate settings, such as corridors, is more likely to occur in times of pressure and poses extra risk for patients. Patients are more likely to have a prolonged wait in the emergency medicine department following a decision for inpatient admission at times of high bed occupancy within the hospital – in winter bed occupancy may frequently exceed 95% [18]. Boarding in the emergency department, waiting for inpatient bed availability, has been shown to be associated with poorer outcomes, including increased length of hospital stay and mortality [19, 20]. Plans for times of pressure should aim to avoid or reduce this practice [12].

Although adaptations for winter pressure undertaken in acute medicine services in the UK have not been previously described, variation in planning for winter has been demonstrated in emergency medicine departments [12]. Previous work has also suggested particular areas where patients may be at higher risk, including where medical patients are treated on non-medical wards [21], where care is provided in hospital corridors rather than appropriate clinical areas [12], or where SDEC facilities are reduced [17]. There is, however, little empirical evidence to support the use of specific planning measures in preference to other measures for medical inpatient services. The heterogeneity in strategies for winter planning that we describe here likely reflects this uncertainty, with no current consensus regarding the most effective way to maintain performance in times of increased service demand.

The results of this survey describe pressure on services that deliver care for urgent medical admissions to acute hospitals within the UK, where increased pressure on services during winter is well recognised [2]. Seasonal variation in demand and in disease is a trend that is seen internationally [8], although excess seasonal mortality is higher in the UK than several European countries, including Germany, Norway and the Netherlands [22, 23].

Survey responses were received from 88.6% of hospitals that took part in SAMBA in 2020, equating to 41.3% of eligible hospitals within the UK [24]. Although this response rate allows identification of variation in the organisational approaches taken to winter pressures in acute medicine services, there may be differences between participating and non-participating hospitals. While the size of hospitals participating in SAMBA is comparable to the acute hospital services nationally [25], covering urban and rural locations across the UK [14], there may have been specific differences in the units that did not participate.

Prior to undertaking this survey, the extent of variation in practice in planning for winter pressures in acute services was not known. We were unable to assess how the adoption of specific planning measures impacted on delivery of patient care within this study due to the nature of the survey. As SAMBA is a single day of care survey, it was not possible to assess trends over time. Longitudinal data may help individual units evaluate the impact of any changes made to mitigate the effect of winter pressures and allow comparison year on year. Factors initially expected to influence variation in adoption of winter planning strategies were assessed here, including hospital size, AMU size, and number of medical admissions per day, however these did not appear to affect the measures chosen, except for redeployment of junior doctors. The underlying reasons for the variation seen here, and the interaction of this variation with the performance of acute care systems at a patient and organisational level, requires further in-depth exploration. The day of care methodology employed within SAMBA cannot provide the analysis of these complex interactions that is needed to fully explain this variation, however these novel findings form a base to guide further study. In order to recommend particular strategies to mitigate the effects of increased periods of pressure, more detailed information is needed on why particular measures were chosen by each unit, and how these impact clinical performance. This may help to expand the evidence base needed to guide winter pressure planning, which is currently lacking.

## Conclusion

Acute internal medicine services are affected by change in demand for services and increased pressure on the service during winter. There is considerable variation in the approach to planning for winter pressure in acute medicine services.

## Data Availability

The datasets used and analysed during the current study are available from the corresponding author on reasonable request.

## References

[1] NHS England (2019). A&E Attendances & Emergency Admission monthly statistics, NHS and independent sector organisations in England.

[2] NHS Improvement (2018). NHS review of winter 2017/18.

[3] NHS England, NHS Improvement (2017). Quick guide: planning for increased seasonal demand in respiratory illness.

[4] Elliot AJ, Cross KW, Fleming DM (2008). Acute respiratory infections and winter pressures on hospital admissions in England and Wales 1990-2005. J Public Health (Oxf).

[5] Vallabhajosyula S, Patlolla SH, Cheungpasitporn W, Holmes DR Jr, Gersh BJ. Influence of seasons on the management and outcomes acute myocardial infarction: An 18-year US study. Clin Cardiol. 2020;43(10):1175-85. 10.1002/clc.23428. 10.1002/clc.23428 PMC7533976 32761957

[6] Ho ATN, Shmelev A, Charbek E (2020). Trends and seasonal variation of hospitalization and mortality of interstitial lung disease in the United States from 2006 to 2016. Respir Res.

[7] Gomes C, Fonseca D, Freitas A. Seasonal variation of diabetes with hyperosmolarity hospitalizations and its characteristics in mainland Portugal. Prim Care Diabetes. 2020;14(5):445-7. 10.1016/j.pcd.2019.12.011. 10.1016/j.pcd.2019.12.011 31937492

[8] Callaly E, Mikulich O, Silke B (2013). Increased winter mortality: the effect of season, temperature and deprivation in the acutely ill medical patient. Eur J Intern Med.

[9] Royal College of Physicians (2007). Acute medical care. The right person, in the right setting – first time. Report for the Acute Medicine Task Force.

[10] NHS England (2015). Improving outcomes for patients with sepsis.

[11] National Institute for Health and Care Excellence (2018). Emergency and acute medical care in over 16s: service delivery and organisation.

[12] Care Quality Commission (2018). Under Pressure: safely managing increased demand in emergency departments.

[13] Holland M, Subbe C, Atkin C, Knight T, Cooksley T, Lasserson D (2020). Society for Acute Medicine Benchmarking Audit 2019 (SAMBA19): trends in acute medical care. Acute Med.

[14] Atkin C, Knight T, Subbe C, Holland M, Cooksley T, Lasserson D (2020). Acute care service performance during winter: report from the winter SAMBA 2020 national audit of acute care. Acute Med.

[15] NHS England, AUEC Review Team and ECIST (2015). Transforming urgent and emergency care services in England.

[16] Health Education England (2018). Supporting winter pressures safely through managed education and training programmes.

[17] Society for Acute Medicine, Royal College of Physicians of Edinburgh (2019). Standards for Ambulatory Emergency Care.

[18] Ewbank L, Thompson J, McKenna H, Anandaciva S. NHS hospital bed numbers: past, present, future. London: The King's Fund; 2020. https://www.kingsfund.org.uk/publications/nhs-hospital-bed-numbers.

[19] Singer AJ, Thode HC, Viccellio P, Pines JM (2011). The association between length of emergency department boarding and mortality. Acad Emerg Med.

[20] Salehi L, Phalpher P, Valani R, Meaney C, Amin Q, Ferrari K (2018). Emergency department boarding: a descriptive analysis and measurement of impact on outcomes. CJEM.

[21] Stylianou N, Fackrell R, Vasilakis C (2017). Are medical outliers associated with worse patient outcomes? A retrospective study within a regional NHS hospital using routine data. BMJ Open.

[22] Laake K, Sverre JM (1996). Winter excess mortality: a comparison between Norway and England plus Wales. Age Ageing.

[23] Healy JD (2003). Excess winter mortality in Europe: a cross country analysis identifying key risk factors. J Epidemiol Community Health.

[24] Royal College of Physicians (2018). Acute internal Medicine - Designing services.

[25] NHS England (2021). Bed availability and occupancy data - overnight.

